# Initiation of Somatostatin analogues for neuroendocrine tumor patients: a cost-effectiveness analysis

**DOI:** 10.1186/s12885-021-08306-5

**Published:** 2021-05-24

**Authors:** Sheila D. Rustgi, Aaron Oh, Jeong Yun Yang, Dasol Kang, Edward Wolin, Chung Y. Kong, Chin Hur, Michelle K. Kim

**Affiliations:** 1 Henry D. Janowitz Division of Gastroenterology, Mount Sinai Health System, Icahn School of Medicine at Mount Sinai, One Gustave L. Levy Place, Box 1118, New York, NY 10029-6574 USA; 2 Columbia University Irving Cancer Center, Columbia University Medical Center, New York, NY USA; 3 Icahn School of Medicine at Mount Sinai, New York, NY USA; 4 Department of Internal Medicine, Lincoln Medical Center, Bronx, NY USA; 5 Division of Hematology and Oncology, Icahn School of Medicine at Mount Sinai, New York, NY USA; 6 Division of General Internal Medicine, Icahn School of Medicine at Mount Sinai, New York, NY USA

**Keywords:** Neuroendocrine tumors, Cost-effectiveness analysis, Somatostatin analogues, Carcinoid, Peptide receptor radionuclide therapy

## Abstract

**Background & Aims:**

Gastroenteropancreatic neuroendocrine tumors (GEP-NETs) are heterogeneous neoplasms. Although some have a relatively benign and indolent natural history, others can be aggressive and ultimately fatal. Somatostatin analogues (SSAs) improve both quality of life and survival for these patients once they develop metastatic disease. However, these drugs are costly and their cost-effectiveness is not known.

**Methods:**

A decision-analytic model was developed and analyzed to compare two treatment strategies for patients with Stage IV GEP-NETs. The first strategy had all patients start SSA immediately while the second strategy waited, reserving SSA initiation until the patient showed signs of progression. Sensitivity analysis was performed to explore model parameter uncertainty.

**Results:**

Our model of patients age 60 with metastatic GEP-NETs suggests empiric initiation of SSA led to an increase 0.62 unadjusted life-years and incremental increase in quality-adjusted life years (QALYs) of 0.44. The incremental costs were $388,966 per QALY and not cost-effective at a willingness-to-pay threshold of $100,000. Death was attributed to GEP-NETs for 94.1% of patients in the SSA arm vs. 94.9% of patients in the DELAY SSA arm. Sensitivity analysis found that the model was most sensitive to costs of SSAs. Using probabilistic sensitivity analysis, the SSA strategy was only cost-effective 1.4% of the time at a WTP threshold of $100,000 per QALY.

**Conclusions:**

Our modeling study finds it is not cost-effective to initiate SSAs at time of presentation for patients with metastatic GEP-NETs. Further clinical studies are needed to identify the optimal timing to initiate these drugs.

## Background

Nearly 200,000 Americans have gastroenteropancreatic neuroendocrine tumors (GEP-NETs), with a higher prevalence than esophageal, gastric, and pancreatic cancers combined [1]. Studies show the incidence is rising over the past 30 years, especially in older adults [2–5]. Nearly half of GEP-NETs patients present with late stage disease from the midgut or pancreas, with 5-year survival ranging from 26 to 75% and substantial associated cancer-related morbidity [2, 6–8]. Treatment is expensive, with estimated costs per patient more than twice that of colon cancer [9–12] in part due to continuous therapy with somatostatin analogues (SSAs), a costly monthly injection [13–18].

For these patients, SSAs are an important treatment that control disease symptoms [19] as well as tumor growth [20–22]. More recently, two randomized placebo-controlled trials (RCTs), the Controlled Study of Lanreotide Antiproliferative Response in Neuroendocrine Tumors (CLARINET) and Placebo-Controlled, Double-Blind, Prospective, Randomized Study on the Effect of Octreotide LAR in the Control of Tumor Growth in Patients With Metastatic Neuroendocrine Midgut Tumors (PROMID) showed a delay in progression of disease for patients treated empirically with SSAs [13, 23–25]. However, consensus guidelines remain unclear as to whether patients should start SSA immediately when diagnosed with metastatic disease, or delay SSA until time of tumor progression [26, 27]. This gap in knowledge leads to physician uncertainty and heterogeneous practice patterns, as well as considerable confusion to patients as to the right course of action to pursue.

The aim of this study is to evaluate the cost-effectiveness of initiating SSAs for patients with metastatic GEP-NETs at time of diagnosis versus at time of disease progression. Modeling is a comparative effectiveness method that allows us to incorporate clinical factors to evaluate the cost-effectiveness of SSAs in metastatic GEP-NETs patients. We hypothesized that, because of the costs, starting SSAs at time of diagnosis may ultimately prolong and improve quality of life, but will not be cost-effective.

## Methods

The study population was patients with metastatic GEP-NETs [28–34]. The goal of this study was to use decision-analytic modeling to help patients and providers choose when to initiate therapy with SSAs. Institutional review board approval was not required for this study that did not use human participants. The data for the model was obtained from published literature and is cited in Table 1.

**Table 1:** Model Parameters

Parameter	Base-Case Value	Sensitivity Analysis	Source
Starting Age	60		13,23
Dose SSA	Octreotide LAR 20 mg/28 days Octreotide 60 mg with PRRT		38–40
**Costs**			
Cost SSA	$4121/month for Octreotide	$2060–$8242	28
Cost PRRT	$205,200	$102,600-410,400	31,41
**Complication, Mortality and Progression Estimates**			
Rate of Complications from SSA	1% over 96 weeks	+/− 25%	13,23
All cause mortality	CDC Life Tables		32
Rate of NET related death, untreated	18 month median survival	+/−25%	25,33
Rate of NET related death, on SSA	39 month median survival	+/−25%	25,33
Rate of NET related death, on PRRT	39 month median survival	+/−25%	37,42
Rate of progression, off SSA	6 month median	+/−25%	13,23
Rate of progression, on SSA	14 month median	+/−25%	13,23
Rate of progression after delaying initiation of SSA	14 month median	+/−25%	24,37
**Utilities**			
Quality of Life pre SSA	0.79	+/−0.1	34,35,43
Quality of Life on SSA	0.79	+/−0.1	34,43
Quality of Life with disease progression	0.72	+/−0.1	34
Quality of Life with uncontrolled symptoms	0.32	+/−0.1	36,44

*SSA* somatostatin analogues; *PRRT* Peptide Receptor Radionuclide Therapy; *NET* neuroendocrine tumor

### Model overview

**Fig. 1:**
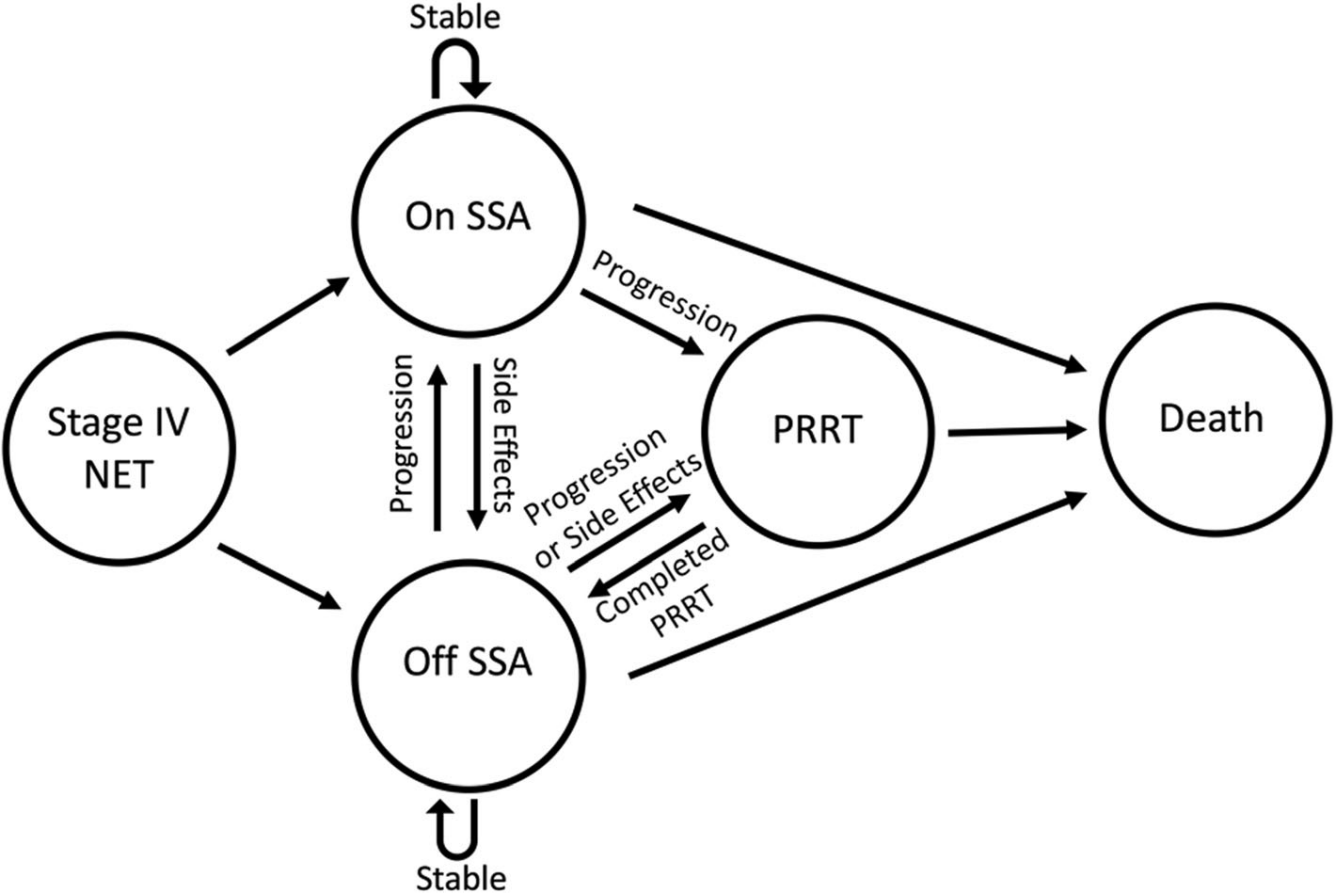
Model Diagram. The figure is a simplified schematic of the Markov model. Circles represent health states and arrows represent transitions. Acronyms: NET = neuroendocrine tumor; SSA = somatostatin analogues; PRRT = Peptide Receptor Radionuclide Therapy

We developed a Markov-based state-transition model using TreeAge Pro (TreeAge 2020, Williamstown, Massachusetts) to compare initiation of SSAs at the time of diagnosis of stage IV GEP-NETs versus waiting to start SSA until the patient’s disease progressed (Fig. 1). The base-case model was based on population characteristics from two prospective RCTs (CLARINET and PROMID) and followed patients from age 60 and cycled monthly until death or age 100 [13, 23–25].

### Management strategy

We simulated and compared two strategies. In the first (SSA), patients were immediately started on SSAs at the time of diagnosis of metastatic GEP-NETs. In the second strategy (DELAY), patients were observed and started on SSAs once their disease progressed. In both strategies, once patients were started on SSAs, if they developed complications from the drug, therapy was discontinued. Alternatively, their disease could progress while on SSA. Once the disease progressed, either on SSA or after stopping SSA due to complications, they were treated with Peptide Receptor Radionuclide Therapy (PRRT). SSA was continued while on PRRT.

### Outcomes

Our outcomes were unadjusted life years gained, incremental costs, quality adjusted life years (QALYs) and the incremental cost-effectiveness ratio (ICER). QALYs are a measure of health which account for both the absolute quantity of survival time adjusted with quality of life. QALYs were subject to half cycle correction and discounted at a standard rate of 3% annually. We assessed cost-effectiveness for each treatment strategy by estimating ICERs. The ICERs were calculated on an efficiency frontier. The willingness-to-pay (WTP) threshold to determine cost-effectiveness was $100,000 per QALY. The optimal timing of whether to delay or initiate therapy right away was defined as the strategy which yielded the highest number of QALYs while having an ICER less than $100,000.

### Model inputs and probabilities

The primary sources of data for model inputs were the two RCTs and the subsequent open label extension studies (Table 1) [13, 23–25]. The starting age for our patient cohort was 60, based on median ages of 62–63 from the two trials. The cost of octreotide, the drug used in the PROMID trials, was $4121 per month [13, 25, 35]. We used octreotide rather than lanreotide based on previous studies suggesting the former is more cost-effective [36, 37]. The cost of PRRT was $51,300 per cycle for a total of four cycles [38]. A 3% discount was applied to costs. The costs were adjusted for inflation to 2020 dollars.

The probability of complications was calculated from serious complication rates requiring cessation of drug use in the CLARINET trial, which was approximately 1%.

All-cause mortality rates were derived using the Center for Disease Control life tables [39]. Because GEP-NETs related deaths do not contribute significantly to the national all-cause mortality rates, the rates were used without adjustment. Rates of GEP-NETs related death both on and off SSA were calculated using survival from Surveillance, Epidemiology and End Results (SEER) studies comparing the pre-SSA era to the post-SSA era [40]. The only new treatment introduced during this time period was SSA. Rates of GEP-NETs related death on PRRT were assumed to be the same as rates of GEP-NETs related death on SSA.

Rates of progression of disease in different scenarios were extrapolated from the two RCTs. Progression rates both on and off SSA were used from the PROMID trial and the extension study. Rates of progression for patients who started SSA after delay of initiation were calculated using the placebo-lanreotide group from the open label extension of CLARINET.

### Quality of life utility values

The utilities for patients with both stable and progressive disease were derived from a recent study that calculated health state utilities from Health Related Quality of Life Questionnaires administered to patients enrolled in the CLARINET trial [41]. These authors found a base case utility value of 0.79 for patients at time of enrollment and found a decrement in utility for patients whose disease progressed (Table 1). The decrement was greater in patients in the placebo arm.

Although carcinoid syndrome has been associated with lower health related quality of life scores, there are no published values for utilities for this health state [42]. Instead, we used utilities from patients with uncontrolled ulcerative colitis symptoms as a proxy, due to frequency of bowel movements and urgency symptoms being similar between the two groups, as was used in a recent cost-effectiveness analysis of telotristat for uncontrolled carcinoid symptoms [42, 43].

### Sensitivity analyses

We conducted one-way deterministic sensitivity analyses and probabilistic sensitivity analysis (PSA) to examine the association of model parameter uncertainty on the modeling results. Sensitivity analyses included varying the costs, utilities and probabilities of progression of disease and probability of GEP-NETs related death (Table 1). For the PSA, we varied all parameter estimates simultaneously with gamma distribution for costs and beta distribution for utilities and probabilities. The PSA was run using Monte Carlo simulations with 100,000 iterations.

## Results

Our base case modeling results showed that starting SSA immediately yielded the greatest life years and QALYs. The SSA strategy increased life expectancy by 0.62 years (7.5 months) but increased total costs by $170,455, due to the longer duration of use of this costly monthly medication (Table 2). The unadjusted life-years with SSA was 4.41 years versus 3.78 years for the DELAY group. However, when accounting for quality of life, starting SSA promptly led to an improvement of 0.44 incremental QALYs. Compared to DELAY, the SSA strategy resulted in an ICER of $388,966 and was not cost-effective at our prescribed WTP threshold of $100,000 per QALY. In the SSA arm, 94.1% of patients died due to GEP-NETs; in contrast, 94.9% of patients died due to GEP-NETs in the DELAY SSA arm.

**Table 2:** Results of Cost-effectiveness Analysis

Strategy	Cost	Incremental Cost	Un adjusted Life-years	Incremental LYs	QALYs	Incremental QALYs	ICER ($/QALY)
SSA	$606,397	$170,455	4.41	0.62^a^	2.90	0.44	$388,966
Delay SSA	$435,942	–	3.78	–	2.46	–	–

^a^Numbers do not sum because of rounding

*SSA* somatostatin analogues; *LY* life years; *QALYs* quality-adjusted life years; *ICER* incremental cost-effectiveness ratio

**Fig. 2:**
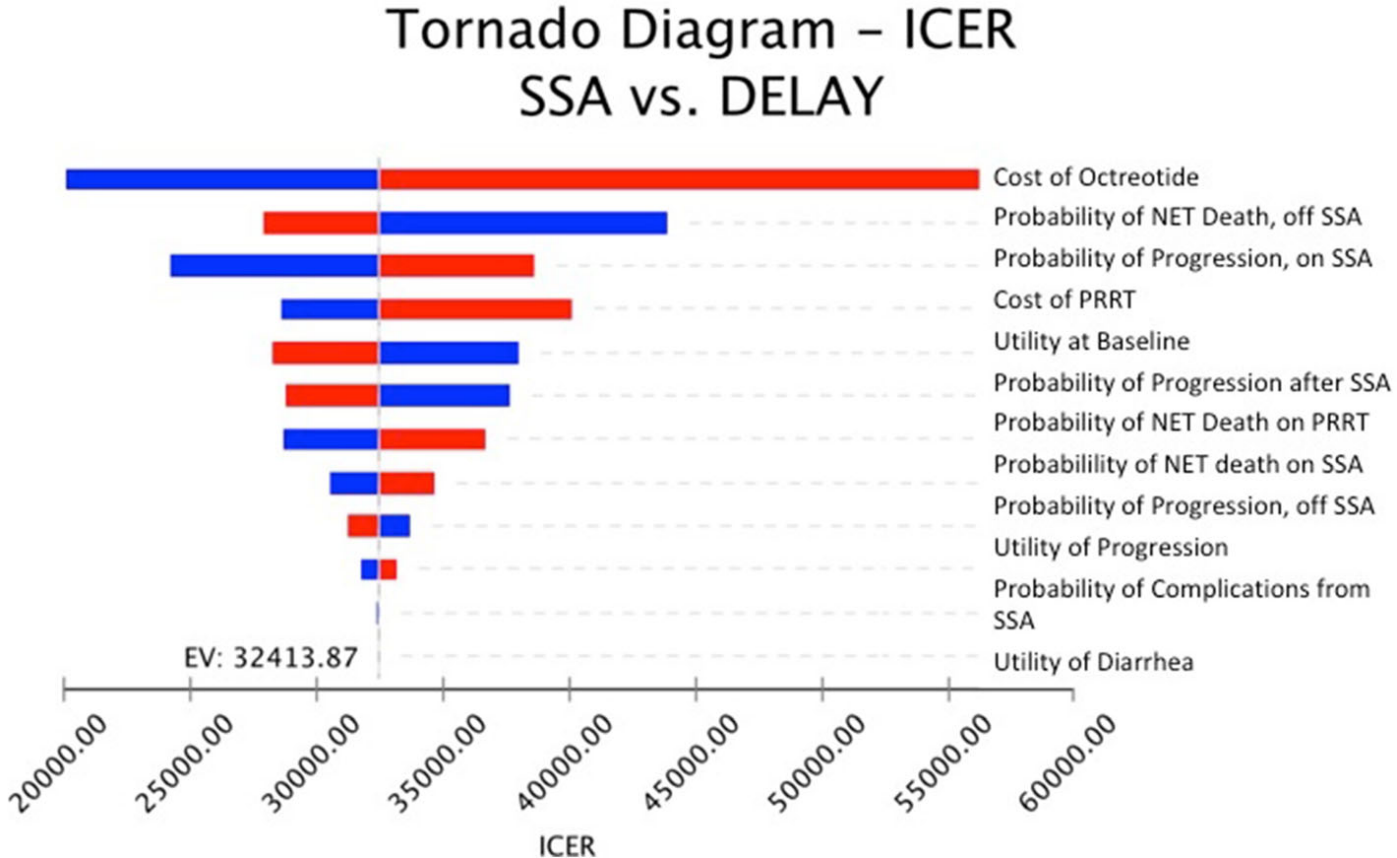
Tornado Diagram – Incremental Cost-Effectiveness Ratio (ICER). The bars indicate the ICERs that result from the range of parameter inputs. NET = Neuroendocrine Tumor; SSA = somatostatin analogues; PRRT = Peptide Receptor Radionuclide Therapy; EV = expected value in base case

The results of our one-way sensitivity analysis showed that the model was most sensitive to the cost of octreotide (Fig. 2). At a range of half to twice the current price, the ICER ranged from $240,517 to $685,864, all above the WTP threshold of $100,000. To meet the WTP threshold of $100,000, the cost of octreotide would have to fall to $110.09. The model was also sensitive to changes in the baseline quality of life, probability of death due to GEP-NETs before starting SSA and the probability of progression of disease while on SSA. In contrast, the quality of life in the setting of uncontrolled diarrhea and risk of complications from SSA, both estimated to be low, did not significantly impact the model. There were no values where the ICER fell below $100,000.

**Fig. 3:**
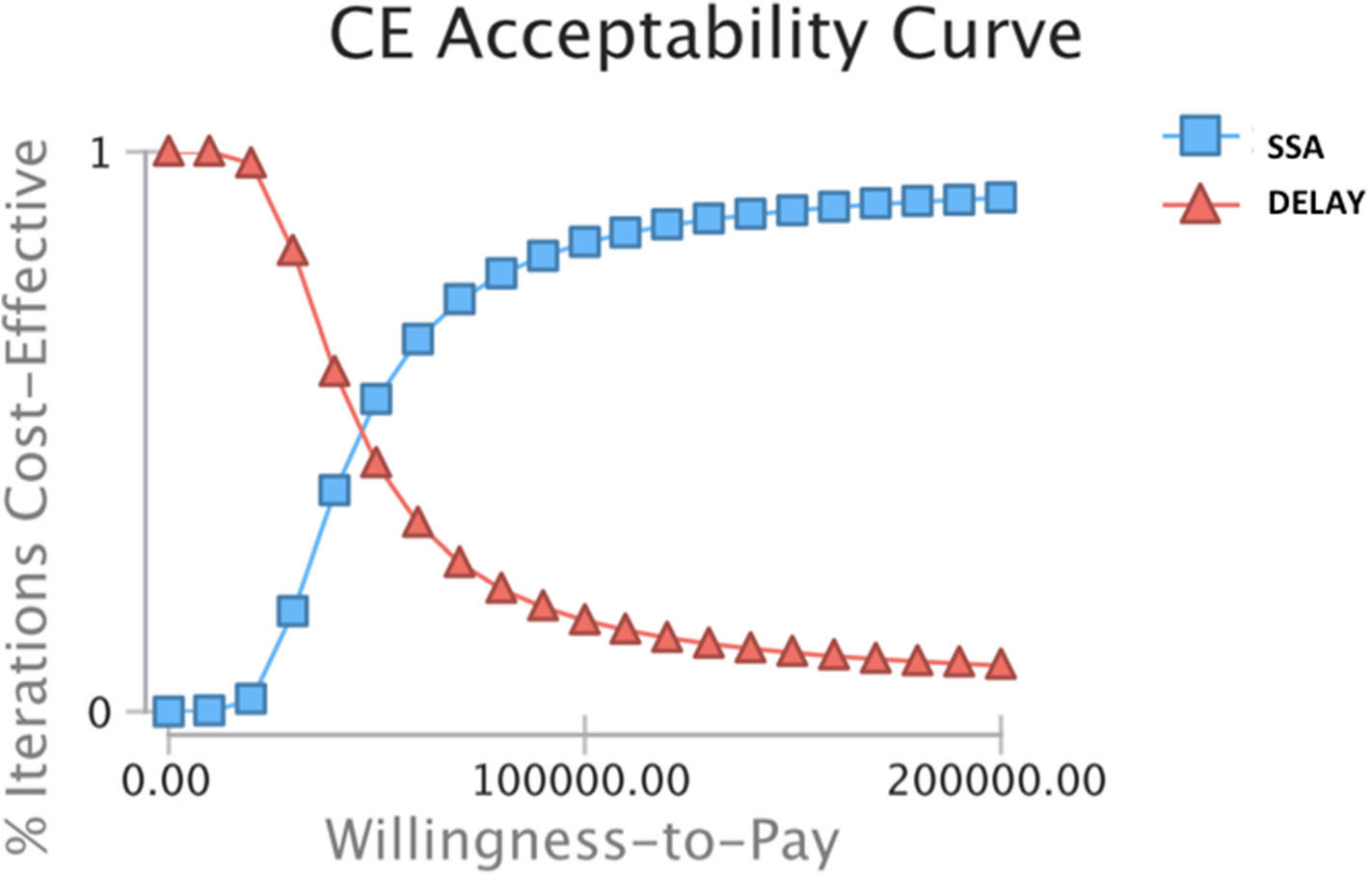
Cost-Effectiveness Acceptability Curve. SSA = somatostatin analogues

The PSA was run for 100,000 simulations. SSA strategy was only cost-effective 1.4% of the time at a WTP threshold of $100,000 per QALY (Fig. 3).

## Discussion

To our knowledge, this is the first cost-effectiveness model that examines when to initiate SSAs for patients with metastatic GEP-NETs. Both the European and American NET societies have issued consensus guidelines stating that initiation of SSAs is up to the treating clinician for patients who are asymptomatic [26, 27]. Our results suggest that although prompt initiation of SSA leads to a slight improvement in unadjusted life years, it is not cost effective when accounting for cost and quality of life.

Although two RCTs have suggested the use of SSA for patients with metastatic GEP-NET leads to delay in tumor progression [13] and improved progression-free survival (PFS) [23], a net mortality benefit was not seen. In the CLARINET trial patients had been observed for 6 months prior to enrollment and had stable disease. A practitioner likely does not know whether the patients’ disease is progressive or stable at the time of evaluation, and so the rates of progression from PROMID were used, where patients were enrolled without a period of observation. Given the expense and recruitment difficulties in conducting large clinical trials, it is unlikely there will be further multi-center RCTs to evaluate the use of SSAs for GEP-NET patients, and thus a microsimulation model may be useful to guide practitioners.

The two strategies in the model were similar, with the main difference being the point at which SSAs was initiated. Because the two strategies were almost identical after starting SSA, the factors that affected patients in the DELAY arm before initiating SSAs (the quality of life before progression of disease and the risk of death before progressing to start SSA) were the most significant factors differentiating the two arms. As discussed earlier, the tornado diagram depicts the factors to which the model is most sensitive (Fig. 2). The costs of the drug can vary widely internationally, and so these results using United States inputs may not be applicable to an international audience.

The results of our model confirm those found in the literature. The unadjusted survival of 4.41 years for the SSA arm is similar to that described in the SEER registry [2, 40]. Although unadjusted life-years for the DELAY group was 3.78 years, this was more than described in SEER data but was plausible given the survival benefit of PRRT, a treatment available in both strategies in the model [44].

Cost-effectiveness models are an important tool to compare treatment costs and to account for the subsequent improvement in quality of life. They are best used as a guide at a societal level to account for benefits to patient care and costs to payors. Limitations of this model include that the two RCTs do not include information on NET-related death and had a selected patient population with low comorbid burdens, and so historical SEER data was used as a proxy. Another limitation is that we only included one medical treatment for patients in whom SSAs were inadequate to control their disease. Although other treatment options such as everolimus, surgery or embolization may be offered in clinical practice, we found no evidence that surgery has different outcomes depending on when SSAs were started, and thus the needs, costs and utilities were unlikely to be different between the two treatment arms and could be omitted from the model. Another limitation is the lack of utility values for quality of life for GEP-NET patients in various treatment stages, and so estimates from other disease states were used. This approximation has been used in other cost-effectiveness studies [42, 43]. However, it does not include approximations for flushing or carcinoid heart disease. Further clinical studies which identify patients most at risk for progression of disease may inform the model and identify a subpopulation in whom empiric initiation of SSA is cost-effective.

## Conclusion

In conclusion, our study uses a well-established method to answer an important clinical question for practitioners caring for GEP-NET patients. Our model found that SSAs are effective and improve QALYS; however, because of the high cost of SSAs, it is not cost-effective to initiate SSA immediately for all patients with metastatic GEP-NETs. Further clinical studies are needed to confirm our findings and the optimal utilization of this drug.

## Data Availability

Not applicable.

## Abbreviations

GEP-NETs: Gastroenteropancreatic neuroendocrine tumors
RCTs: Randomized Controlled Trials
CLARINET: Controlled Study of Lanreotide Antiproliferative Response in Neuroendocrine Tumors
PROMID: Placebo-Controlled, Double-Blind, Prospective, Randomized Study on the Effect of Octreotide LAR in the Control of Tumor Growth in Patients With Metastatic Neuroendocrine Midgut Tumors
SSA: Somatostatin Analogues
PRRT: Peptide Receptor Radionuclide Therapy
QALY: Quality Adjusted Life Year
WTP: Willingness to Pay
